# Continuous Theta-Burst Stimulation on the Left Posterior Inferior Frontal Gyrus Perturbs Complex Syntactic Processing Stability in Mandarin Chinese

**DOI:** 10.1162/nol_a_00140

**Published:** 2024-06-24

**Authors:** Junjie Wu, Yao Cheng, Xingfang Qu, Tianmin Kang, Yimin Cai, Peng Wang, Emiliano Zaccarella, Angela D. Friederici, Gesa Hartwigsen, Luyao Chen

**Affiliations:** Key Research Base of Humanities and Social Sciences of the Ministry of Education, Academy of Psychology and Behavior, Tianjin Normal University, Tianjin, China; Max Planck Partner Group, School of International Chinese Language Education, Beijing Normal University, Beijing, China; Department of Psychology, Skidmore College, Saratoga Springs, NY, USA; Institute of Psychology, University of Regensburg, Regensburg, Germany; Institute of Psychology, University of Greifswald, Greifswald, Germany; Department of Neuropsychology, Max Planck Institute for Human Cognitive and Brain Sciences, Leipzig, Germany; Lise Meitner Research Group Cognition and Plasticity, Max Planck Institute for Human Cognitive and Brain Sciences, Leipzig, Germany; Wilhelm Wundt Institute for Psychology, Leipzig University, Leipzig, Germany; Institute of Educational System Science, Beijing Normal University, Beijing, China

**Keywords:** Chinese, continuous theta burst stimulation (cTBS), inferior frontal gyrus (IFG), language, syntactic processing

## Abstract

The structure of human language is inherently hierarchical. The left posterior inferior frontal gyrus (LpIFG) is proposed to be a core region for constructing syntactic hierarchies. However, it remains unclear whether LpIFG plays a causal role in syntactic processing in Mandarin Chinese and whether its contribution depends on syntactic complexity, working memory, or both. We addressed these questions by applying inhibitory continuous theta-burst stimulation (cTBS) over LpIFG. Thirty-two participants processed sentences containing embedded relative clauses (i.e., complex syntactic processing), syntactically simpler coordinated sentences (i.e., simple syntactic processing), and non-hierarchical word lists (i.e., word list processing) after receiving real or sham cTBS. We found that cTBS significantly increased the coefficient of variation, a representative index of processing stability, in complex syntactic processing (esp., when subject relative clause was embedded) but not in the other two conditions. No significant changes in *d*′ and reaction time were detected in these conditions. The findings suggest that (a) inhibitory effect of cTBS on the LpIFG might be prominent in perturbing the complex syntactic processing stability but subtle in altering the processing quality; and (b) the causal role of the LpIFG seems to be specific for syntactic processing rather than working memory capacity, further evidencing their separability in LpIFG. Collectively, these results support the notion of the LpIFG as a core region for complex syntactic processing across languages.

## INTRODUCTION

The structure of human language is inherently hierarchical (e.g., Berwick & Chomsky, 2016; Everaert et al., 2015; Friederici, 2017; Hauser et al., 2002). Consider, for example, the sentence “Tom who met Mary knew John.” It is “Tom” who “knew John,” not “Mary,” even though the linear distance between “Mary” and “knew” is much shorter than that between “Tom” and “knew.” Structurally, the relative clause “who met Mary” is center-embedded between the subject “Tom” and the main verb “knew” in the main clause, with “Tom” and “knew” being structurally closer (Bulut et al., 2018; O’Grady, 1997; Santi & Grodzinsky, 2010), thus demonstrating the hierarchical nature of human language. The construction of such a complex sentence/hierarchical structure involves the recursive application of a fundamental syntactic operation known as *merge*, which combines two elements into a new constituent each time it is applied (Chomsky, 1995; Fujita, 2014; Goucha et al., 2017; Hoshi, 2018, 2019; Miyagawa et al., 2013; Zaccarella et al., 2017).

**Figure 1.**
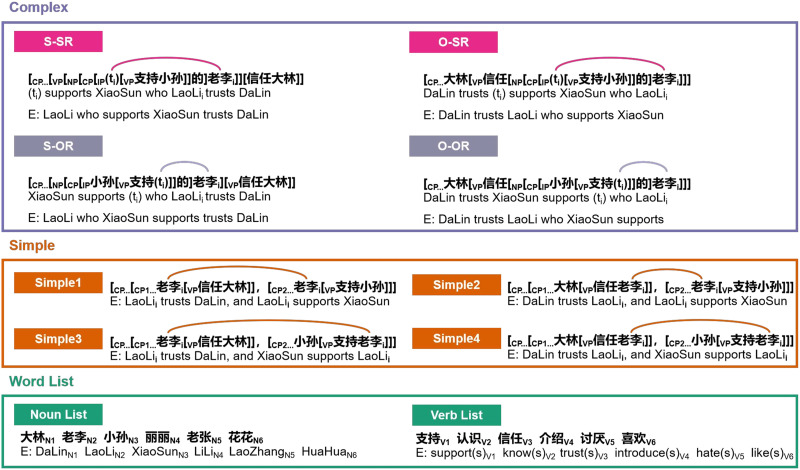
Sequence processing conditions with example sentences/word lists. *Complex* (syntactic processing condition) refers to the presentation of complex sentences with subject or object relative clauses embedded in the object (O-SR/O-OR) and subject (S-SR/S-OR) positions of the main clauses. As illustrated, a verb phrase (VP) is center-embedded between the trace (t) and the target noun (N) as co-indexed by the subscript “i” in SR (the dependency of t_i_ and N_i_ was marked by a pink arc), leading to a structurally more complex structure than in OR (the dependency of t_i_ and N_i_ was marked by a purple arc). *Simple* (syntactic processing conditions) refers to the presentation of coordinated sentences, in which the co-indexed nouns were labeled with the subscript “i,” and their dependencies were highlighted by the orange arcs. Each simple sentence semantically corresponds to the complex sentence at the same position (e.g., Simple1 is semantically the same to S-SR) in this figure. *Abbreviations*: CP = complementizer phrase; IP = inflection phrase. English translations (E) were provided. *Word list* (verbal working memory conditions) contains Noun List and Verb List, which are free of hierarchical structure.

Scrutinizing the neural substrates of merge, numerous neurolinguistic studies converged on the notion that the left posterior inferior frontal gyrus (LpIFG), particularly the left Brodmann Area (BA) 44 within Broca’s area, might be critical for merge, or more generally, syntactic processing (Chen et al., 2021; Chen et al., 2023; Goucha & Friederici, 2015; Makuuchi et al., 2009; Maran, Friederici, et al., 2022; Ohta et al., 2013; Schell et al., 2017; Wang et al., 2021; Wu et al., 2019; Zaccarella et al., 2017; Zaccarella et al., 2021; Zaccarella & Friederici, 2015). Previous studies (e.g., Kroczek et al., 2019; Kuhnke et al., 2017; Meyer et al., 2018; Sakai et al., 2002; van der Burght et al., 2023) have primarily examined languages with rich morphological variations, such as German and Japanese, leaving it is unknown whether the findings related to the LpIFG can be generalized to syntactic processes at large. Recently, the LpIFG was proposed to be engaged in the syntactic processes of various topologically distinct languages, such as Mandarin Chinese (e.g., Chang et al., 2020; Chen et al., 2023; Wu et al., 2019; Zhu et al., 2022). Mandarin Chinese is a structurally left-branching language (cf. Figure 1 in Materials) that lacks morphosyntactic information and is heavily meaning-dependent, in stark contrast to other languages which are rich in morphological changes (Chao, 1968; Zhu, 1985). Therefore, Mandarin Chinese might be a valuable case to investigate whether LpIFG’s involvement pertains specifically to morphologically complex languages or extends to general syntactic hierarchical processing (independent of the language typological differences). In addition, most of the above-mentioned previous studies utilized functional magnetic resonance imaging (fMRI) to reveal correlative structure–function relationships. However, the causal relevance of LpIFG for syntactic processes remains largely unclear (Blank & Fedorenko, 2017; Buchsbaum et al., 2005; Diachek et al., 2020; Fedorenko et al., 2011; Hickok et al., 2003; Santi & Grodzinsky, 2007b, 2010).

Moreover, the extent to which the function of LpIFG is specific to syntax or domain-general cognitive mechanisms (such as working memory) remains controversial (Grodzinsky & Santi, 2008; Kaan & Swaab, 2002; Makuuchi et al., 2009; Makuuchi et al., 2013; Rogalsky et al., 2008). For instance, Makuuchi et al. (2009) and Makuuchi et al. (2013) found that LpIFG (particularly pars opercularis) responds to structural complexity during sentence processing, while activity in the left inferior frontal sulcus (LIFS) was linked to the processing of the dependency length, reflecting working memory load. Nevertheless, Rogalsky and Hickok (2011) assumed that sentences with multiple-embedded clauses still require increased working memory capacity. Based on individual functional localizers, Fedorenko et al. (2011) identified a language-specific network, in which only the LpIFG (containing both BA 45 and BA 44) responded to the contrast of language > non-word list. Despite the finer functional parcellation of the LpIFG, these areas also overlapped with a domain-general multiple-demand network that supports a variety of non-linguistic cognitive tasks (Blank & Fedorenko, 2017; Diachek et al., 2020). Non-linguistic cognitive tasks seemed to either partially overlap with or surround BA 45 and BA 44, leading to the claim that “Broca’s area is not a natural kind” (Fedorenko & Blank, 2020). Consequently, it remains unclear whether LpIFG is causally relevant for syntactic processing, working memory, or both. To address this question, we added a verbal working memory task to assess the relationship between LpIFG and working memory by comparing participants’ performance on the tasks after real and sham brain stimulations.

Across the last decades, as an effective noninvasive brain stimulation technique, transcranial magnetic stimulation (TMS) has increasingly been used to probe causal structure–function relationships with a high spatial resolution (e.g., Hallett, 2000; Hartwigsen, 2015; Hartwigsen & Silvanto, 2023; Qu et al., 2022; Uddén et al., 2017). Several studies have investigated the causal role of LpIFG with various syntactic tasks, as summarized in Table 1. It shows that TMS over LpIFG induced diverging behavioral changes in syntactic processing, ranging from facilitation (e.g., Sakai et al., 2002; Uddén et al., 2008; van der Burght et al., 2023) to inhibition (e.g., Carreiras et al., 2012; Ishkhanyan et al., 2020; Maria-Korina et al., 2015; Meyer et al., 2018; Uddén et al., 2017). It is noteworthy that these studies adopted various behavioral indices and their sensitivities also varied. Processing quality and stability are two important dimensions in language processing (e.g., Lim & Godfroid, 2015; Segalowitz & Hulstijn, 2005; Segalowitz & Segalowitz, 1993). Specifically, *d*′ serves as a reliable indicator of processing quality (Pinet & Nozari, 2021) because it reflects the ability to discriminate between signal and noise (Stanislaw & Todorov, 1999) and provides deeper insights than mere accuracy rates (Kuhl et al., 2005; Tolentino & Tokowicz, 2014). Moreover, reaction time (RT) is utilized as a processing quality measure due to its direct assessment of response speed to stimuli (Buccino et al., 2005; Gough et al., 2005), providing an immediate gauge of cognitive processing and capturing the impact of TMS (Qu et al., 2022). Additionally, the coefficient of variation (CV) is considered to reflect the degree of automation as it measures response variation—with less variation suggesting greater stability and automation (Lim & Godfroid, 2015; Segalowitz & Hulstijn, 2005; Segalowitz & Segalowitz, 1993).

**Table 1.** Summary of previous TMS studies targeting the left parietal IFG during syntactic processing

Study	Language	Tasks	TMS protocol (types, timing, frequencies, intensities, pulse number)	Stimulation sites (coordinates)	Indices	Results
Sakai et al. (2002)	Japanese	Syntactic decision task	event-related TMS, online, 55%–98% AMT, paired pulses	Left IFG: x = −63 ± 1.1, y = 11 ± 5.7, z = 15 ± 4.4	ΔRT	Left F3op/F3t: a reduction of RT (i.e., smaller Δ*RT*) in explicit syntactic decisions.
		Semantic decision task		Left MFG: x = 42 ± 4.0, y = 25 ± 4.5, z = 48 ± 3.5		Left F2: null effects.
Uddén et al. (2008)	Artificial grammar	Implicit acquisition task	rTMS, offline, 1 Hz, 110% RMT, biphasic pulse	Left and right BA44/45: x = ± 48, y = 16, z = 20	Endorsement rate, *d*-prime (*d*′), RT	Left BA44/45: shorter RT.
		Classification task				Bilateral BA44/45: larger rejection rate of non-grammatical items.
Carreiras et al. (2012)	Spanish	Grammaticality judgment task	rTMS, online, 10 Hz, 45% of maximum stimulator output for Broca’s area, 60% of maximum output for right intraparietal sulcus	Left BA44: x = −58, y = 12, z = 22	RT, AccR	Broca’s area (left BA44): TMS pulses improved RTs in grammatical trials and AccR in ungrammatical trials, and also reduced the agreement effect.
				Right IPS: x = 40, y = −48, z = 40		
Acheson and Hagoort (2013)	Dutch	Sentence reading task	cTBS, offline, 50 Hz, 41% of the stimulator output mean AMT, 600 pulses	Left MTG: x = −52, y = −50, z = −8	Total reading time, looking times, first fixation, duration	Left IFG and left MTG: stimulation modulated the ambiguity effect for total reading times in the temporarily ambiguous sentence region relative to the control group.
				Left IFG: x = −44, y = 0, z = 22		
Maria-Korina et al. (2015)	Greek	Syntactic language task	rTMS, online, 0.3 Hz, 45% stimulus intensity, 5 pulses	Broca’s area	ΔRT	Δ*RT*s between syntactic normal sentences and syntactic abnormal sentences for the syntactic task and Δ*RT*s between abnormal sentences for both tasks (SynT-SemT) were close to significant differences.
		Semantic language task				
Kuhnke et al. (2017)	German	Sentence comprehension task	rTMS, online, 10 Hz, 90% RMT, biphasic pulse	Left posterior IFG: x = 54, y = 14, z = 13	Drift-diffusion model parameters (esp., Δ drift rates)	Left posterior IFG: significantly increased performance decline (lower drift rate) for object-first sentences with long-distance dependencies.
				Left PT: x = −42, y = −40, z = 10		
Uddén et al. (2017)	Artificial grammar	Implicit acquisition task	rTMS, offline, 1 Hz, 110% RMT, continuous biphasic pulse train	Left inferior frontal cortex (BA 44/45): x = −48, y = 16, z = 20	Endorsement rate, RT	Left BA44/45: endorsement rate reduced.
		Classification task				
Meyer et al. (2018)	German	The audio-visual sentence processing task	rTMS, online, 12.5 Hz, 90% RMT, 5 pulses	Left IFG: x = −53, y = 7, z = 22	RT, *d*-prime (*d*′), *β*	Left IFG: termination bias increased significantly (i.e., *β* was more negative).
				Right IFG: x = 55, y = 7, z = 19		
Kroczek et al. (2019)	German	Lexical decision task	rTMS, online, 10 Hz, 90% RMT, 3 pulses	Left posterior IFG: x = −60, y = 12, z = 16	RT, AccR; Δ*μ*V	RT of high-cloze sentence endings was shorter than for low-cloze sentences, and RT of correct sentences was shorter than for incorrect ones.
				Left posterior STG/STS: x = −50, y = 42, z = 2		At the mid-sentence verb: TMS over LpIFG: a 200 ms post-verb onset frontal positivity; TMS over left posterior STG/STS: parietal negativity at 200–400 ms post verb onset.
Coetzee et al. (2023)	English	Reasoning task	cTBS, offline, 50 Hz, 80% AMT, 600 pulses	Left BA44: x = −50, y = 18, z = 18	RT, Δ AccR	Broca’s area (left BA44) (left) and left medial BA8: significant differences in percent accuracy change for linguistic and logic reasoning. The cTBS to BA44 reduced the AccR of linguistic reasoning and grammaticality judgment task, but cTBS to medial BA8 and left TOS improved the AccR of linguistic reasoning and grammaticality judgment task.
		Grammaticality judgment task		Left medial BA8: x = −6, y = 40, z = 38		
				Left TOS: x = −25, y = 85, z = 25		
Maran, Numssen, et al. (2022)	German	Audiovisual grammaticality judgment task	rTMS, online, 10 Hz, 90% RMT, 5 pulses	Left BA44: x = −48, y = 17, z = 16	RT, AccR, mean amplitude of the ESN, EEG signal (P600)	Null results. TMS did not affect the generation of the ESN (prediction error, according to a predictive coding perspective), nor late repairing processes (late positivity/P600).
				Left SPL: x = −34, y = −42, z = 70		
van der Burght et al. (2023)	German	Sentence completion task	rTMS, online, 10 Hz, 90% RMT, 5 pulses	Left BA44: x = −51, y = 11, z = 14	RT, AccR	Left posterior IFG (BA 44/45): an overall decrease in AccR.
				Left BA45: x = −51, y = 33, z = 2		

*Note*. The endorsement rate is defined as the number of sequences classified as grammatically independent of their actual status, divided by the total number of recorded responses for each factor level (Uddén et al., 2017). *Abbreviations*: TMS = transcranial magnetic stimulaton; IFG = inferior frontal gyrus; RT = Reaction Time; MFG = middle frontal gyrus; AMT = active motor threshold; rTMS = repetitive TMS; BA = Brodmann Area; RMT = resting motor threshold; AccR = Accuracy rate; IPS = intraparietal sulcus; cTBS = continuous theta-burst stimulation; MTG = middle temporal gyrus; PT = planum temporale; STG = superior temporal gyrus; STS = superior temporal sulcus; TOS = transverse occipital sulcus; SPL = superior parietal lobe; ESN = Early Syntactic Negativity.

Regardless of the directions of such modulations, LpIFG seems to be causally relevant for syntactic processes mainly in languages with abundant morphological changes, such as German or Japanese. Moreover, artificial grammar learning or processing studies implied a ubiquitous role of the LpIFG across languages (Uddén et al., 2008, 2017). However, several issues remain unaddressed: First, the *functional specificity* of LpIFG in syntactic tasks requires clarification through the inclusion of tasks from other domains, such as working memory tasks. Second, it is still debated whether LpIFG responds to structured sequences regardless of their level of *structural complexity* (Petersson et al., 2012; Uddén et al., 2017), or if syntactic complexity matters as a moderator, as hypothesized by a prominent neurolinguistic model (Friederici, 2011, 2017) that links BA 44 in the LpIFG with complex syntactic processing. Third, although previous fMRI studies suggested that LpIFG might be a *critical syntactic region* across topologically distinct languages (Chen et al., 2023; Friederici, 2017; Hammer et al., 2007; Maran, Friederici, et al., 2022; Maran, Numssen, et al., 2022), it is unknown whether LpIFG plays a causal role in Mandarin Chinese syntactic processing, or is simply co-activated due to the features (i.e., heavily meaning-dependent and impoverished morphosyntactic cues) of Mandarin Chinese.

To ascertain whether the LpIFG exhibits a causal relationship with the hierarchical processing of general syntax, we need to clarify whether this relationship exists and is independent of verbal working memory and language type. Therefore, we combined TMS before task processing (offline, using the well-established inhibitory continuous theta burst stimulation (cTBS) protocol; Huang et al., 2005) with a subsequent syntactic processing paradigm in Mandarin Chinese adapted from Liu et al. (2023; see Materials section for details), in which the syntactic complexity, as well as the working memory load, were manipulated. We hypothesize that the LpIFG plays a causal role for syntactic processing regardless of language type and working memory. If this holds true, we would expect that cTBS over LpIFG would significantly affect the processing of Mandarin Chinese sentences with higher syntactic complexities, leading to inhibited behavioral performances (i.e., reduced response qualities and/or increased processing instability), independent of the working memory effects.

## MATERIALS AND METHODS

### Participants

Thirty-two healthy adult Chinese native speakers were recruited in this experiment (15 males and 17 females; age: 19.7 ± 1.3 years; see Behavioral Data Analyses of the Whole Set of Participants in the Supporting Information, available at https://doi.org/10.1162/nol_a_00140, for more details). All participants were right-handed with normal or corrected-to-normal vision. None of them reported a history of psychiatric or neurological diseases and presented any potential contradictions against cTBS. Each participant signed the written informed consent and was reimbursed 60 ¥ (CNY) per hour after completing the whole experiment. This study met the guidelines of the Declaration of Helsinki and was approved by the local ethics committee.

### Materials

Syntactic complexity was manipulated by three conditions: complex sentences with embedded relative clauses (i.e., the complex syntactic processing condition), simple coordinated sentences, and non-mergeable word lists. Complex sentences included either subject relative clause (SR) or object relative clause (OR) embeddings at both subject and object positions of the main clause. Crucially, as illustrated in Figure 1, in Mandarin Chinese SR is structurally more complex than OR due to the fact that SR contains a longer dependency between the trace (t) and the head noun (a verb phrase is center-embedded) in a non-canonical word order (VOS; see also Hsiao & Gibson, 2003). Thus SR was proposed to be more difficult to process (Chen et al., 2008; Hsiao & Gibson, 2003; Sun et al., 2016; Xu et al., 2020a, 2020b; Yang et al., 2010). The simple sentences also contained four sub-types according to the co-reference dependencies as shown in Figure 1. Additionally, the word list condition required participants not only to access the words but also to recall and match their position within each list, drawing on working memory resources. The word list condition thus served as a working memory control condition.

The materials utilized in the present study (Figure 1) were adapted from Liu et al. (2023; see Supporting Information for details). In brief, considering the duration of aftereffects of cTBS (∼40 min; Huang et al., 2005), each session contained 36 trials per condition (i.e., complex syntactic processing, simple syntactic processing, and word-list processing), with half of them being incorrect. The complex syntactic processing condition included sentences with either subject-relative clauses or object-relative clauses embedded (18 sentences for each type). The direct comparison of subject and object relative clauses was of no interest in this study. Lexical semantics were controlled for by using identical content words (nouns and verbs) across these conditions, and sentence-level/thematic meanings (who did what to whom) were also similar between complex and simple sentences, with the only variation being in syntactic complexity of the sentences (see also Bulut et al., 2018; Indefrey et al., 2004; Just et al., 1996; Thibault et al., 2021; Xu et al., 2020a, 2020b, for similar designs). Besides, word frequencies as well as the occurrences of the single words and word pairs (such as a bigram composed of a noun and a verb or of two nouns/verbs) were carefully controlled so that participants were unable to make a response by a particular word or a word pair after reading each sequence (i.e., a sentence or a word list). Bigrams of nouns or verbs of the word lists were also checked to exclude potentially mergeable pairs. Therefore, especially for the syntactic processing conditions, non-syntactic strategies could not be applied as also confirmed by the previous study of Liu et al. (2023). The sentence and word-list tokens were different between the sessions.

### Procedures

#### Main procedures

**Figure 2.**
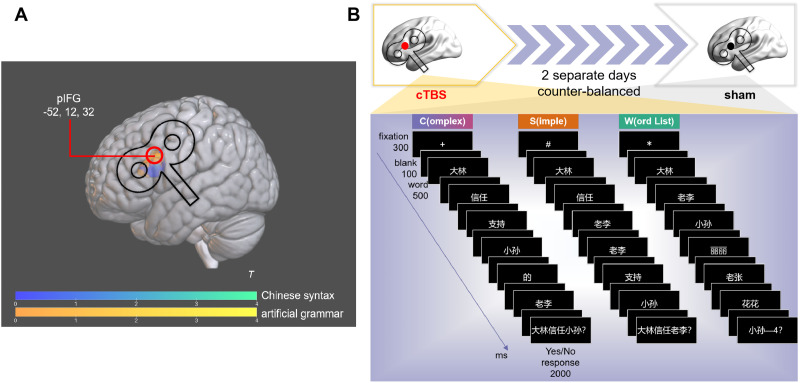
(A) The predefined stimulation site from two studies in MNI coordinates (see Procedures for details). (B) Experimental procedure with the specific timing parameters for each condition.

Given that the effects of TMS can last up to 50 min (Wischnewski & Schutter, 2015), within-subject designs are commonly utilized in TMS research (e.g., Sakai et al., 2002; Schuhmann et al., 2009; Sliwinska et al., 2021; Uddén et al., 2017; Ward et al., 2022), which typically involves participants completing the task across two separate visits. In addition, according to a previous meta-study, the within-subject design showed greater statistical power than the between-subject design in the TMS studies (Qu et al., 2022). Therefore, we opted for a within-subject design in the present study. Specifically, participants underwent two sessions, an effective and a sham (placebo) cTBS session, on two separate days to minimize potential carry-over effects. (The cTBS effect was assumed to last for about 40 min at maximum; Huang et al., [2005].) The session order was counterbalanced across participants. For the syntactic processing conditions, participants were required to judge whether the probing sentences correctly reflect the contents (i.e., “Who did what to whom?”) of the test sentences, whereas, for the word-list processing condition, participants had to judge whether the position and probing word matched correctly for each trial. All sequences from these conditions were pseudorandomized and visually presented in a slide-by-slide fashion with the same timing parameters (Figure 2) using E-prime 2.0 (Psychology Software Tools, Inc., Pittsburgh, PA, USA; https://support.pstnet.com). Trials of the same condition began with a specific fixation type to minimize condition-switching load and help participants adapt to the tasks on time (see also Matchin et al., 2017). The tasks in each session lasted approximately 20 min.

#### Continuous theta-burst stimulation

Before the actual experiment, participants’ high-resolution T1-weighted images were acquired via a 3-T MRI Scanner (Siemens Prisma) for subsequent TMS neuronavigation. Individual anatomical data were obtained for co-registration with the following imaging parameters: repeated time (TR) = 2,530 ms; echo time (TE) = 2.98 ms; flip angle = 7°; field-of-view (FOV) = 256 × 256 mm; matrix size = 256 × 256 mm; in-plane resolution within slices = 1.0 × 1.0 mm; slice thickness = 1.00 mm; number of slices = 192.

During the cTBS session, we used a frameless stereotaxic system (Localite GmbH, Bonn, Germany) to monitor coil placement. The group stimulation site was predefined by two recent fMRI studies. Chen et al. (2023) adopted a jabberwocky sentence processing paradigm to scrutinize the neural underpinnings of Mandarin Chinese syntactic processing, in which content words were replaced by pseudo-words with the lexical-semantics deprived, and the real Mandarin Chinese function-word-based syntactic structures were retained. They identified the activation of LpIFG at the whole-brain level under the contrast of structure > word list and suggested that this region might be shared in Chinese syntactic processing as a key syntactic region. Intriguingly, a recent artificial grammar processing study using Chinese-like pseudo-words observed that the construction of syntactic hierarchies at the basic level of merge, guided by artificial syntactic rules, also activated LpIFG. The signal intensity in this region was significantly correlated with performance on complex sentence processing (i.e., sentences with relative clauses embedded as used in the present study) in Mandarin Chinese (Liu et al., 2023). Hence, the mean peak activation coordinates (MNI: x = −52, y = 12, z = 32) were extracted from the intersection results of the LpIFG activation between these two studies as the standard *target site of syntax* for cTBS in the present study (Figure 2A).

Each participant’s anatomical image was loaded into the navigation system and manually registered with the identification of the anterior and posterior commissures, as well as the point on the falx to localize precise target stimulation sites. The participant-specific sites were indexed by the trajectory markers using the MNI coordinate system. An MRI co-registration procedure was conducted to map the 3D model from the standard MNI space to real individual space for each participant. A headband with reflective spherical markers tracked by the navigation system was worn by the participants, which would guide the placement of the coil over the target site for each individual. The angles of the markers were checked and adjusted to be orthogonal to the skull during TMS navigation.

A TMS stimulator (MagPro X100, MagVenture) with a standard 70 mm figure-of-eight coil (MagVenture MFC-B65) was used for stimulation. Before administering TMS, participants’ resting motor threshold (RMT) was determined. We delivered single pulses of TMS over the motor cortex of the left hemisphere until distinct motor-evoked potentials were observed from the relaxed first dorsal interosseous muscle in the right-hand using electromyography. RMT was defined as the lowest stimulation intensity producing a visible motor-evoked potential of approximately 50 *μ*V (peak-to-peak amplitude) on at least 5 out of 10 consecutive trials (Steel et al., 2016). Participants’ RMT ranged from 38% to 74% of the maximum stimulator output, with a mean threshold of 56% (standard deviation [*SD*] = 9.6%). cTBS was then applied to LpIFG, with triplets of TMS pulses at 50 Hz being delivered at 5 Hz, resulting in a 40 s train of 600 pulses in total (Hellriegel et al., 2012; Huang et al., 2005; Steel et al., 2016). Considering that RMT has a higher intensity than active motor threshold (Chen et al., 1998; Fried et al., 2019), we opted to use 80% of RMT in our study to ensure an adequate level of intensity (see also Jung & Lambon Ralph, 2021; Qu et al., 2022; Steel et al., 2016). Sham stimulation was performed by flipping the coil over with the settings of cTBS.

We have to acknowledge that, although we attempted to implement a single-blind procedure in our study, most of our participants (29/32) were able to correctly identify the real stimulation on a questionnaire after the second TMS session. This was due to the fact that stimulation over the inferior frontal gyrus inevitably stimulates facial muscles and nerves, which may cause discomfort or pain to participants. This challenge has been encountered in many previous studies (e.g., Hartwigsen et al., 2010; Jodzio et al., 2023; Pestalozzi et al., 2018). Nevertheless, we believe that calculating the difference between the data from real and sham stimulation and comparing the difference between conditions (see next section for details) may help mitigate this issue. To ensure the validity of the results, an independent experimenter without access to the condition labels reanalyzed the data. This independent reanalysis yielded similar results, providing additional confidence in the reliability of the findings (see Supporting Information for more details). In addition, the potential impact of session order was tested by including a group factor (we divided the subjects into two groups, based on the session order of real and sham cTBS) in our mixed models (see Supporting Information for more details).

### Behavioral Data Analyses

Data analyses were performed in JASP 0.17.1.0 (JASP Team, 2023; https://jasp-stats.org/). Following the seminal study of Sakai et al. (2002), the behavioral change (Δ), calculated by effective − sham cTBS of each condition, was calculated for the following behavioral indices:

(a)  To assess the processing quality, that is, whether participants’ responses were sensitive and fast enough to correctly respond to the signal, *d-prime* (*d*′) and *RT* were calculated (see also Meyer et al., 2018). Specifically, *d*′ was calculated using the following formula: z-transform (hit rate: correct response attempts/total target attempts when set correctly) − z-transform (false alarm rate: incorrect response attempts/total target attempts when set incorrectly). In situations where the hit rate or false alarm rate was equal to 1 or 0, which makes the calculation of the Z-scores problematic, we adjusted the hit or false alarm attempts by adding 0.5, and also increased the total target attempts setting by 1 (Stanislaw & Todorov, 1999). Additionally, RT directly assesses the response speed to stimuli, which was calculated by only averaging the response latency on correctly responded trials.
(b)  To assess the processing stability, the *CV* was calculated based on RT (Segalowitz & Segalowitz, 1993): *CV* = *SD*/mean *RT*. This index was proposed to be a reliable and robust measure of automatization in language learning and processing (e.g., Lim & Godfroid, 2015; Segalowitz & Hulstijn, 2005; Segalowitz & Segalowitz, 1993).

Here, we deemed both *d*′ and *RT* as processing quality indices, and *CV* as the response state index, thus separating the behavioral indices into two dimensions. It should be noted that the RT-related indices were selectively analyzed for correct responses, and trials with RTs shorter than 150 ms were removed in advance for each participant (see also Maran, Numssen, et al., 2022). If necessary, outliers of the behavioral changes for each index were interpolated by *Q*1 − 1.5 *IQR* or *Q*3 + 1.5 *IQR*, respectively (quantile [Q]; interquartile range [IQR]). For each index, the behavioral changes were tested against 0 by one-sample *t* tests to evaluate whether cTBS was able to induce a significant change for a particular condition. Thereafter, one-way repeated measures analyses of variance were performed to test the behavioral change differences in the three (complex syntactic, simple syntactic, and word list) and the four (SR, OR, simple syntactic, and word list) processing conditions for each behavioral index. For each analysis of a certain index, the *p* values of the one-sample *t* tests were Bonferroni-corrected. Furthermore, as for the comparison of the four conditions, since the number of trials of SR/OR processing condition should be lower than the number of trials of simple syntactic/word list processing condition (originally 18 trials for SR/OR vs. 36 trials for each of the other two conditions), Spearman correlation tests were performed first to evaluate whether the differences in the number of trials Δ*trial*(*s*) would be correlated with the behavioral change differences between these conditions. For example, if the SR processing condition was compared with simple syntactic processing condition, the behavioral change difference (such as the Δ*CV* difference = Δ*CV*_*SR*_ − Δ*CV*_*simple*_) as well as the difference in the number of correctly responded trials (Δ*trial* = *trial*_*SR*_ − *trial*_*simple*_) would be calculated, and then the Spearman correlation test would be performed between Δ*CV*_*SR*_ − Δ*CV*_*simple*_ and Δ*trial*. If any correlation was significant, the Δ*trial* would be then treated as a covariate and regressed out.

## RESULTS

**Figure 3.**
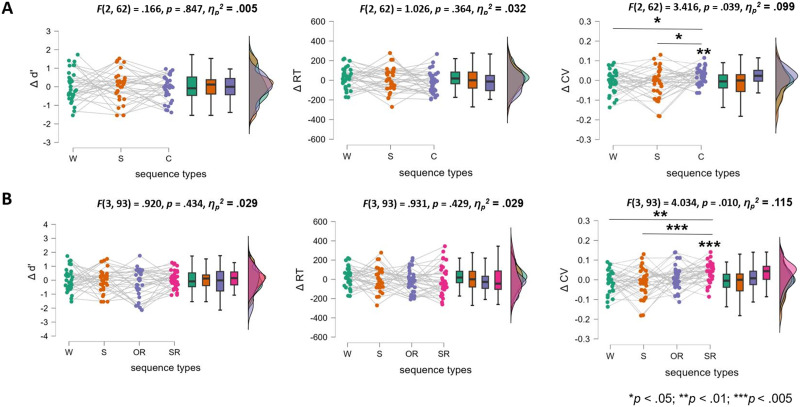
Results for conditions. (A) Behavioral results for the three conditions. (B) Behavioral analysis results for the four conditions. *Abbreviations*: C = complex syntactic processing (colored in purple); SR = complex sentence with subject relative clause embedded processing (colored in red); OR = complex sentence with object relative clause embedded processing (colored in purple); S = simple syntactic processing (colored in orange); W = word list processing (colored in green).

We did not observe any trials with responses shorter than 150 ms. A descriptive summary of the behavioral results is provided in Table 2. As shown in Figure 3A, Δ*CV* revealed a significant behavioral change for the complex syntactic processing condition (higher Δ*CV* than 0: *t*(31) = 3.292, *p*_bonf_ = 0.006, Cohen’s *d* = 0.582), but not for the other two conditions (simple syntactic processing: Δ*CV* ∼ 0: *t*(31) = −0.945, *p*_bonf_ = 1.000, Cohen’s *d* = –0.167; word list processing: Δ*CV* ∼ 0: *t*(31) = −0.798, *p*_bonf_ = 1.000, Cohen’s *d* = −0.141). Significant behavioral change differences among complex syntactic, simple syntactic, and word list processing conditions could also be found in Δ*CV* (*F*(2, 62) = 3.416, *p* = 0.039, *η*_*p*_^2^ = 0.099). Post-hoc paired-samples *t* tests showed that the Δ*CV* for complex syntactic processing was larger than those of the other two conditions (simple syntactic processing: *t*(31) = 2.401, *p* = 0.019, Cohen’s *d* = 0.619; word list processing: *t*(31) = 2.096, *p* = 0.040, Cohen’s *d* = 0.540). There was no significant difference between the word list and the simple syntactic processing conditions (*t*(31) = 0.305, *p* = .333, Cohen’s *d* = 0.079).

**Table 2.** Summary of the behavioral data

Conditions	Δ*d*′		Δ*RT*		Δ*CV*	
	*M*	*SD*	*M*	*SD*	*M*	*SD*
All	−0.073	0.669	−15.777	111.841	0.024	0.042
C, OR	−0.243	1.036	−23.735	116.062	0.011	0.062
SR	0.097	0.647	−8.807	153.258	0.037	0.051
S	0.011	0.835	4.749	121.728	−0.013	0.076
W	0.048	0.828	23.197	102.77	−0.008	0.056

*Note*. *Abbreviations*: *d*′ = *d*-prime; RT = reaction time; CV = coefficient of variation; C = complex syntactic processing condition; OR = complex sentence with object relative clause embedded processing condition; SR = complex sentence with subject relative clause embedded processing condition; S = simple syntactic processing condition; W = word list processing condition.

Furthermore, as shown in Figure 3B, when the complex syntactic processing condition was split into the OR and SR processing conditions, Δ*V* showed a significant difference from 0 particularly for the SR processing condition (higher Δ*V* than 0: *t*(31) = 4.135, *p*_bonf_ = 0.003, Cohen’s *d* = 0.731), but not for the OR processing condition (Δ*V* ∼ 0: *t*(31) = 1.034, *p*_bonf_ = 1.000, Cohen’s *d* = 0.183). Δ*V* also showed significant differences in the four conditions (i.e., OR, SR, simple syntactic, and word list processings; *F*(3, 93) = 4.034, *p* = 0.010, *η*_*p*_^2^ = 0.115). According to the post-hoc paired-samples *t* test results, the Δ*V* of the SR processing condition was much larger than that of the simple syntactic processing condition (*t*(31) = 3.124, *p* = 0.004, Cohen’s *d* = 0.805) as well as of the word list processing condition (*t*(31) = 2.831, *p* = 0.009, Cohen’s *d* = 0.729). Nevertheless, the Δ*V* of the OR processing condition could not be statistically differentiated from the other three conditions (0 > *t*s(31) ≥ −1.207, *p*s ≥ 0.647, 0 > Cohen’s *d*s ≥ −0.311). It is also noteworthy that Δ*trials* were not significantly correlated with the Δ*V* differences in the conditions. In particular, the Δ*V* differences between SR and the other two (simple syntactic/word list processing) conditions could not be accounted for by the differences in the number of trials (SR & simple: *rho* = 0.169, *p* = 0.354; SR & word list: *rho* = 0.105, *p* = 0.568). And the null Δ*V* differences between OR and the other two conditions could not be explained by the unbalanced number of trials which might result in the lack of statistic power (OR & simple: *rho* = −0.080, *p* = 0.663; OR & word list: *rho* = 0.069, *p* = 0.707). Therefore, for the comparison of the four conditions, the differences in the number of trials were unlikely to affect the results.

No differences among either the three (i.e., complex syntactic, simple syntactic, and word list processing) or the four conditions (i.e., OR, SR, simple syntactic, and word list processing) could be found for Δ*d*′ and Δ*RT* (see Figure 3 for the statistics).

These results indicate that after cTBS, the complex syntactic processing presented more RT variation and became more unstable for decision-making.

## DISCUSSION

The present study explored the causal role of LpIFG in syntactic processing in Mandarin Chinese with inhibitory cTBS. Results showed that for the complex syntactic processing condition, especially for the condition of processing the most complex sentences with subject-relative clauses embedded, increased processing instability was observed on the basis of Δ*CV*, while no significant changes could be detected for the processing quality indices (i.e., *d*′ and *RT*).

Numerous previous studies proposed that LpIFG might constitute a core region for merge/syntactic processing (e.g., Chen et al., 2021; Chen et al., 2023; Goucha & Friederici, 2015; Indefrey et al., 2004; Makuuchi et al., 2009; Makuuchi et al., 2013; Maran, Friederici, et al., 2022; Musso et al., 2003; Ohta et al., 2013; Wang et al., 2021; Zaccarella & Friederici, 2015; Zhu et al., 2022). In line with these investigations, our study further elucidated the specific contribution of LpIFG, demonstrating a key role for complex syntactic processing in Mandarin Chinese, but not for simple syntax or working memory. This was evidenced by cTBS-induced variations in processing stability for the complex syntactic processing condition. The observed specific inhibitory effect of cTBS on syntactic complexity converges with a series of artificial grammar learning/processing studies in which complex grammars increased activation of LpIFG (especially BA 44) compared to simpler ones (Chen et al., 2019; Chen et al., 2023; Petersson et al., 2012). Likewise, syntactic complexity was manipulated by various approaches such as word order scrambling (Goucha & Friederici, 2015; Matchin et al., 2017; Ohta et al., 2013; Pallier et al., 2011; Tyler et al., 2010), syntactic movement (Cooke et al., 2002; Fiebach et al., 2005; Grodzinsky, 2000; Rogalsky et al., 2008; Santi & Grodzinsky, 2007a, 2007b), and multiple syntactic embedding (den Ouden et al., 2012; Makuuchi et al., 2009; Makuuchi et al., 2013; Pallier et al., 2011; Wang et al., 2021) in natural language materials. These previously also demonstrated significant activation of LpIFG for increasing syntactic complexity (see also Friederici, 2017, for a systematic review). A recent TMS study in German (Kuhnke et al., 2017) further observed that TMS over the LpIFG impaired the object-first non-canonical sentence processing condition only (i.e., the syntactically more difficult condition). Moreover, when LpIFG was perturbed by TMS, German native speakers had difficulties in chunking words into longer (i.e., syntactically more complex) phrases (Meyer et al., 2018). These findings suggested a causal role of LpIFG in complex syntactic processing, which is consistent with the present results.

Moreover, in our study, given the relatively lower syntactic complexity which did not require a high involvement of LpIFG, no significant changes for the simple syntactic processing condition after cTBS could be observed. The working memory task of the word list processing condition was more challenging than the simple syntactic processing condition, and its cognitive demands were assumed to be comparable with the complex syntactic processing condition, as demonstrated by Liu et al. (2023). Yet, word list processing performance was not impaired by TMS, supporting the idea that the syntactic role of the LpIFG should be independent of the working memory capacity (Bornkessel et al., 2005; Fiebach et al., 2002;  Fiebach et al., 2005; Makuuchi et al., 2009; Makuuchi et al., 2013; Meyer et al., 2012).

As a convenient protocol for stimulating the brain for a relatively short period (∼40 s), cTBS has been utilized in several recent studies to establish the causal link between the neural activity of LpIFG and syntactic processing (Acheson & Hagoort, 2013; Coetzee et al., 2023). However, significant stimulation effects appeared in different behavioral indices of different syntactic tasks. For instance, no accuracy differences but differences in eye-tracking indices could be found during a syntactic ambiguity resolution task after cTBS (Acheson & Hagoort, 2013), whereas accuracy was significantly decreased for a grammaticality judgment task after cTBS to LpIFG (Coetzee et al., 2023). In our study, neither Δ*d*′ nor Δ*RT* showed statistical differences in the conditions. However, with respect to the response state (i.e., how to process the sequences), changes in the processing stability (i.e., measured by Δ*CV*) revealed robust inhibitory cTBS effects on LpIFG selectively for the complex syntactic (esp., SR) processing (sub-)condition. On the one hand, it should be noted that the transient perturbation caused by cTBS is not equivalent to a structural lesion which might lead to a significant functional loss or impairment of the target region, disabling the successful completion of the tasks (see also Hartwigsen et al., 2013; Huang et al., 2005). On the other hand, demonstrating the causal role of LpIFG is, by no means, speaking against the functional importance of the other regions serving as critical nodes of the syntactic network (e.g., Chang et al., 2020; Chen et al., 2021; Chen et al., 2023; Chou et al., 2012; den Ouden et al., 2012; Friederici & Gierhan, 2013; Humphries et al., 2005; Rogalsky & Hickok, 2009; Sun et al., 2021; Wang et al., 2008; Wu et al., 2019; Xu et al., 2020a). Functional compensation for the short-lived disruption of LpIFG was speculated to take place even within hundreds of milliseconds during online TMS (Maran, Numssen, et al., 2022), let alone the 40 s offline cTBS. Therefore, it is not surprising that no qualitative behavioral changes (e.g., the decrease in accuracy) were detected by the present study, even though the processing state showed inhibitory effects. Furthermore, we are cautious of making a null result claim without exploring the potential indices synthetically/comprehensively. Future studies utilizing cTBS or other noninvasive brain stimulation protocols are encouraged to develop more sensitive indices (either behavioral or neurocognitive) and tasks to systematically evaluate the causal role of LpIFG in syntactic processing.

However, our results might shed limited light on the debate regarding the role of LpIFG in syntax and domain-general hierarchical processing. It is plausible to hypothesize that non-linguistic domains like music and behavior share cognitive and neural resources with syntax, given the similarity of their hierarchical systems to those in linguistic domains (Coopmans et al., 2023; Fitch & Martins, 2014; Fujita, 2014; Pulvermüller & Fadiga, 2010; Stout & Chaminade, 2009). Nevertheless, neuroimaging studies suggest only a limited overlap between linguistic and non-linguistic hierarchical processing in the LpIFG (Fazio et al., 2009; Friederici, 2020; Roy et al., 2013; Thibault et al., 2021). This finding leads us to propose that syntax serves as a distinct core computational mechanism within language hierarchies. This uniqueness may stem from linguistic constraints such as the notion that every word carries a syntactic word category label (e.g., noun, verb), suggesting that syntax-specific hierarchies are exclusive to language and may not extend to other cognitive domains (Berwick et al., 2013; Moro, 2014; Zaccarella et al., 2021). Therefore, future investigations should employ specialized experimental designs to further examine the LpIFG’s causal role in hierarchical processing across various domains.

In summary, we provide the first evidence for a causal role of LpIFG in complex syntactic processing in Mandarin Chinese from the perspective of processing stability in healthy young adults. This finding is also consistent with the majority of studies on morphologically rich languages, suggesting that LpIFG is sensitive to general syntactic hierarchical processing. Moreover, our results converge on the notion that syntactic processing is also independently housed in LpIFG in Mandarin Chinese (e.g., Chen et al., 2023; Zhu et al., 2022), which is a core syntactic region, regardless of language types and working memory, causally backing up the human language faculty (Hauser et al., 2002).

## ACKNOWLEDGMENTS

We would like to sincerely thank Yannan Ji, Qianming Liu, Hongfu Qu, Qiwen Cheng, and Yuxin Yang for their assistance in TMS operation and data collection during the extremely hard and dangerous period of COVID-19. Special thanks are extended to Yuming Ke for her assistance in data preprocessing, and to Liping Feng for the constructive inputs. Besides, this work is in memory of our fallen loved ones who suffered deeply from the pandemic.

## FUNDING INFORMATION

Luyao Chen, National Social Science Fund of China (https://dx.doi.org/10.13039/501100012456), Award ID: 22CYY017.

## AUTHOR CONTRIBUTIONS

**Junjie Wu**: Data curation, Formal analysis, Investigation, Writing – original draft, Writing – review & editing. **Yao Cheng**: Data curation, Formal analysis, Investigation, Writing – original draft, Writing – review & editing. **Xingfang Qu**: Data curation, Formal analysis, Investigation, Writing – original draft, Writing – review & editing. **Tianmin Kang**: Writing – review & editing. **Yimin Cai**: Writing – review & editing. **Peng Wang**: Writing – review & editing. **Emiliano Zaccarella**: Writing – review & editing. **Angela D. Friederici**: Writing – review & editing. **Gesa Hartwigsen**: Writing – review & editing. **Luyao Chen**: Conceptualization, Funding acquisition, Supervision, Writing – original draft, Writing – review & editing.

## DATA AND CODE AVAILABILITY STATEMENT

Data analyses were performed in JASP 0.17.1.0 (JASP Team, 2023; https://jasp-stats.org/), and the data for reproducing the presented behavioral analyses are available at: https://osf.io/x9mzs/.

## Supplementary Material



## TECHNICAL TERMS

Merge: A fundamental syntactic operation combining two syntactic objects (X, Y) into a new set {X, Y} for hierarchical processing.
Coefficient of variation (CV): The degree of automation/stability as it measures response variation reflected by reaction time: *CV* = *SD*/*M* RT.
Continuous theta-burst stimulation (cTBS): Refers to the inhibitory transcranial magnetic stimulation with triplets of TMS pulses at 50 Hz delivered at 5 Hz.

## References

[bib200] References marked with an asterisk appear in Table 1.

[bib1] *Acheson, D. J., & Hagoort, P. (2013). Stimulating the brain’s language network: Syntactic ambiguity resolution after TMS to the inferior frontal gyrus and middle temporal gyrus. Journal of Cognitive Neuroscience, 25(10), 1664–1677. 10.1162/jocn_a_00430, 23767923

[bib2] Berwick, R. C., & Chomsky, N. (2016). Why only us: Language and evolution. MIT Press. 10.7551/mitpress/9780262034241.001.0001

[bib3] Berwick, R. C., Friederici, A. D., Chomsky, N., & Bolhuis, J. J. (2013). Evolution, brain, and the nature of language. Trends in Cognitive Sciences, 17(2), 89–98. 10.1016/j.tics.2012.12.002, 23313359

[bib4] Blank, I. A., & Fedorenko, E. (2017). Domain-general brain regions do not track linguistic input as closely as language-selective regions. Journal of Neuroscience, 37(41), 9999–10011. 10.1523/JNEUROSCI.3642-16.2017, 28871034 PMC5637122

[bib5] Bornkessel, I., Zysset, S., Friederici, A. D., von Cramon, D. Y., & Schlesewsky, M. (2005). Who did what to whom? The neural basis of argument hierarchies during language comprehension. NeuroImage, 26(1), 221–233. 10.1016/j.neuroimage.2005.01.032, 15862222

[bib6] Buccino, G., Riggio, L., Melli, G., Binkofski, F., Gallese, V., & Rizzolatti, G. (2005). Listening to action-related sentences modulates the activity of the motor system: A combined TMS and behavioral study. Cognitive Brain Research, 24(3), 355–363. 10.1016/j.cogbrainres.2005.02.020, 16099349

[bib7] Buchsbaum, B. R., Olsen, R. K., Koch, P., & Berman, K. F. (2005). Human dorsal and ventral auditory streams subserve rehearsal-based and echoic processes during verbal working memory. Neuron, 48(4), 687–697. 10.1016/j.neuron.2005.09.029, 16301183

[bib8] Bulut, T., Cheng, S.-K., Xu, K.-Y., Hung, D. L., & Wu, D. H. (2018). Is there a processing preference for object relative clauses in Chinese? Evidence from ERPs. Frontiers in Psychology, 9, Article 995. 10.3389/fpsyg.2018.00995, 30038589 PMC6046449

[bib9] *Carreiras, M., Pattamadilok, C., Meseguer, E., Barber, H., & Devlin, J. T. (2012). Broca’s area plays a causal role in morphosyntactic processing. Neuropsychologia, 50(5), 816–820. 10.1016/j.neuropsychologia.2012.01.016, 22285905

[bib10] Chang, C. H. C., Dehaene, S., Wu, D. H., Kuo, W.-J., & Pallier, C. (2020). Cortical encoding of linguistic constituent with and without morphosyntactic cues. Cortex, 129, 281–295. 10.1016/j.cortex.2020.04.024, 32535379

[bib11] Chao, Y. R. (1968). A grammar of spoken Chinese. University of California Press.

[bib12] Chen, B., Ning, A., Bi, H., & Dunlap, S. (2008). Chinese subject-relative clauses are more difficult to process than the object-relative clauses. Acta Psychologica, 129(1), 61–65. 10.1016/j.actpsy.2008.04.005, 18538740

[bib13] Chen, L., Gao, C., Li, Z., Zaccarella, E., Friederici, A. D., & Feng, L. (2023). Frontotemporal effective connectivity revealed a language-general syntactic network for Mandarin Chinese. Journal of Neurolinguistics, 66, Article 101127. 10.1016/j.jneuroling.2023.101127

[bib14] Chen, L., Goucha, T., Männel, C., Friederici, A. D., & Zaccarella, E. (2021). Hierarchical syntactic processing is beyond mere associating: Functional magnetic resonance imaging evidence from a novel artificial grammar. Human Brain Mapping, 42(10), 3253–3268. 10.1002/hbm.25432, 33822433 PMC8193521

[bib15] Chen, L., Wu, J., Fu, Y., Kang, H., & Feng, L. (2019). Neural substrates of word category information as the basis of syntactic processing. Human Brain Mapping, 40(2), 451–464. 10.1002/hbm.24386, 30240492 PMC6865558

[bib16] Chen, R., Tam, A., Bütefisch, C., Corwell, B., Ziemann, U., Rothwell, J. C., & Cohen, L. G. (1998). Intracortical inhibition and facilitation in different representations of the human motor cortex. Journal of Neurophysiology, 80(6), 2870–2881. 10.1152/jn.1998.80.6.2870, 9862891

[bib17] Chomsky N. (1995). The minimalist program. MIT Press. 10.7551/mitpress/9780262527347.001.0001

[bib18] Chou, T.-L., Lee, S.-H., Hung, S.-M., & Chen, H.-C. (2012). The role of inferior frontal gyrus in processing Chinese classifiers. Neuropsychologia, 50(7), 1408–1415. 10.1016/j.neuropsychologia.2012.02.025, 22414592

[bib19] *Coetzee, J. P., Johnson, M. A., Lee, Y., Wu, A. D., Iacoboni, M., & Monti, M. M. (2023). Dissociating language and thought in human reasoning. Brain Sciences, 13(1), Article 67. 10.3390/brainsci13010067, 36672048 PMC9856203

[bib20] Cooke, A., Zurif, E. B., DeVita, C., Alsop, D., Koenig, P., Detre, J., Gee, J., Pinãngo, M., Balogh, J., & Grossman, M. (2002). Neural basis for sentence comprehension: Grammatical and short-term memory components. Human Brain Mapping, 15(2), 80–94. 10.1002/hbm.10006, 11835600 PMC6872024

[bib21] Coopmans, C. W., Kaushik, K., & Martin, A. E. (2023). Hierarchical structure in language and action: A formal comparison. Psychological Review, 130(4), 935–952. 10.1037/rev0000429, 37166848

[bib22] den Ouden, D.-B., Saur, D., Mader, W., Schelter, B., Lukic, S., Wali, E., Timmer, J., & Thompson, C. K. (2012). Network modulation during complex syntactic processing. NeuroImage, 59(1), 815–823. 10.1016/j.neuroimage.2011.07.057, 21820518 PMC3195988

[bib23] Diachek, E., Blank, I., Siegelman, M., Affourtit, J., & Fedorenko, E. (2020). The domain-general multiple demand (MD) network does not support core aspects of language comprehension: A large-scale fMRI investigation. Journal of Neuroscience, 40(23), 4536–4550. 10.1523/JNEUROSCI.2036-19.2020, 32317387 PMC7275862

[bib24] Everaert, M. B. H., Huybregts, M. A. C., Chomsky, N., Berwick, R. C., & Bolhuis, J. J. (2015). Structures, not strings: Linguistics as part of the cognitive sciences. Trends in Cognitive Sciences, 19(12), 729–743. 10.1016/j.tics.2015.09.008, 26564247

[bib25] Fazio, P., Cantagallo, A., Craighero, L., D’Ausilio, A., Roy, A. C., Pozzo, T., Calzolari, F., Granieri, E., & Fadiga, L. (2009). Encoding of human action in Broca’s area. Brain, 132(7), 1980–1988. 10.1093/brain/awp118, 19443630

[bib26] Fedorenko, E., Behr, M. K., & Kanwisher, N. (2011). Functional specificity for high-level linguistic processing in the human brain. Proceedings of the National Academy of Sciences of the United States of America, 108(39), 16428–16433. 10.1073/pnas.1112937108, 21885736 PMC3182706

[bib27] Fedorenko, E., & Blank, I. A. (2020). Broca’s area is not a natural kind. Trends in Cognitive Sciences, 24(4), 270–284. 10.1016/j.tics.2020.01.001, 32160565 PMC7211504

[bib28] Fiebach, C. J., Schlesewsky, M., & Friederici, A. D. (2002). Separating syntactic memory costs and syntactic integration costs during parsing: The processing of German WH-questions. Journal of Memory and Language, 47(2), 250–272. 10.1016/S0749-596X(02)00004-9

[bib29] Fiebach, C. J., Schlesewsky, M., Lohmann, G., von Cramon, D. Y., & Friederici, A. D. (2005). Revisiting the role of Broca’s area in sentence processing: Syntactic integration versus syntactic working memory. Human Brain Mapping, 24(2), 79–91. 10.1002/hbm.20070, 15455462 PMC6871727

[bib30] Fitch, W. T., & Martins, M. D. (2014). Hierarchical processing in music, language, and action: Lashley revisited. Annals of the New York Academy of Sciences, 1316(1), 87–104. 10.1111/nyas.12406, 24697242 PMC4285949

[bib31] Fried, P., Jannati, A., Morris, T., Buss, S., Santarnecchi, E., Shafi, M., & Pascual-Leone, A. (2019). Relationship of active to resting motor threshold influences the aftereffects of theta-burst stimulation. Brain Stimulation, 12(2), 465. 10.1016/j.brs.2018.12.513

[bib32] Friederici, A. D. (2011). The brain basis of language processing: From structure to function. Physiological Reviews, 91(4), 1357–1392. 10.1152/physrev.00006.2011, 22013214

[bib33] Friederici, A. D. (2017). Language in our brain: The origins of a uniquely human capacity. MIT Press. 10.7551/mitpress/9780262036924.001.0001

[bib34] Friederici A. D. (2020). Hierarchy processing in human neurobiology: How specific is it? Philosophical Transactions of the Royal Society of London Series B: Biological Sciences, 375(1789), Article 20180391. 10.1098/rstb.2018.0391, 31735144 PMC6895560

[bib35] Friederici, A. D., & Gierhan, S. M. E. (2013). The language network. Current Opinion in Neurobiology, 23(2), 250–254. 10.1016/j.conb.2012.10.002, 23146876

[bib36] Fujita, K. (2014). Recursive merge and human language evolution. In T. Roeper & M. Speas (Eds.), Recursion: Complexity in cognition (pp. 243–264). Springer. 10.1007/978-3-319-05086-7_11

[bib37] Goucha, T., & Friederici, A. D. (2015). The language skeleton after dissecting meaning: A functional segregation within Broca’s area. NeuroImage, 114, 294–302. 10.1016/j.neuroimage.2015.04.011, 25871627

[bib38] Goucha, T., Zaccarella, E., & Friederici, A. D. (2017). A revival of *Homo loquens* as a builder of labeled structures: Neurocognitive considerations. Neuroscience and Biobehavioral Reviews, 81(B), 213–224. 10.1016/j.neubiorev.2017.01.036, 28318539

[bib39] Gough, P. M., Nobre, A. C., & Devlin, J. T. (2005). Dissociating linguistic processes in the left inferior frontal cortex with transcranial magnetic stimulation. Journal of Neuroscience, 25(35), 8010–8016. 10.1523/JNEUROSCI.2307-05.2005, 16135758 PMC1403818

[bib40] Grodzinsky, Y. (2000). The neurology of syntax: Language use without Broca’s area. Behavioral and Brain Sciences, 23(1), 1–71. 10.1017/S0140525X00002399, 11303337

[bib41] Grodzinsky, Y., & Santi, A. (2008). The battle for Broca’s region. Trends in Cognitive Sciences, 12(12), 474–480. 10.1016/j.tics.2008.09.001, 18930695

[bib42] Hallett, M. (2000). Transcranial magnetic stimulation and the human brain. Nature, 406(6792), 147–150. 10.1038/35018000, 10910346

[bib43] Hammer, A., Goebel, R., Schwarzbach, J., Münte, T. F., & Jansma, B. M. (2007). When sex meets syntactic gender on a neural basis during pronoun processing. Brain Research, 1146, 185–198. 10.1016/j.brainres.2006.06.110, 16904083

[bib44] Hartwigsen, G. (2015). The neurophysiology of language: Insights from non-invasive brain stimulation in the healthy human brain. Brain and Language, 148, 81–94. 10.1016/j.bandl.2014.10.007, 25468733

[bib45] Hartwigsen, G., Baumgaertner, A., Price, C. J., Koehnke, M., Ulmer, S., & Siebner, H. R. (2010). Phonological decisions require both the left and right supramarginal gyri. Proceedings of the National Academy of Sciences of the United States of America, 107(38), 16494–16499. 10.1073/pnas.1008121107, 20807747 PMC2944751

[bib46] Hartwigsen, G., Saur, D., Price, C. J., Ulmer, S., Baumgaertner, A., & Siebner, H. R. (2013). Perturbation of the left inferior frontal gyrus triggers adaptive plasticity in the right homologous area during speech production. Proceedings of the National Academy of Sciences of the United States of America, 110(41), 16402–16407. 10.1073/pnas.1310190110, 24062469 PMC3799383

[bib47] Hartwigsen, G., & Silvanto, J. (2023). Noninvasive brain stimulation: Multiple effects on cognition. Neuroscientist, 29(5), 639–653. 10.1177/10738584221113806, 35904354

[bib48] Hauser, M. D., Chomsky, N., & Fitch, W. T. (2002). The faculty of language: What is it, who has it, and how did it evolve? Science, 298(5598), 1569–1579. 10.1126/science.298.5598.1569, 12446899

[bib49] Hellriegel, H., Schulz, E. M., Siebner, H. R., Deuschl, G., & Raethjen, J. H. (2012). Continuous theta-burst stimulation of the primary motor cortex in essential tremor. Clinical Neurophysiology, 123(5), 1010–1015. 10.1016/j.clinph.2011.08.033, 21982298

[bib50] Hickok, G., Buchsbaum, B., Humphries, C., & Muftuler, T. (2003). Auditory–motor interaction revealed by fMRI: Speech, music, and working memory in area Spt. Journal of Cognitive Neuroscience, 15(5), 673–682. 10.1162/jocn.2003.15.5.673, 12965041

[bib52] Hoshi, K. (2018). Merge and labeling as descent with modification of categorization: A neo-Lennebergian approach. Biolinguistics, 12, 39–54. 10.5964/bioling.9135

[bib53] Hoshi, K. (2019). More on the relations among categorization, merge and labeling, and their nature. Biolinguistics, 13, 1–21. 10.5964/bioling.9147

[bib51] Hsiao, F., & Gibson, E. (2003). Processing relative clauses in Chinese. Cognition, 90(1), 3–27. 10.1016/S0010-0277(03)00124-0, 14597268

[bib54] Huang, Y.-Z., Edwards, M. J., Rounis, E., Bhatia, K. P., & Rothwell, J. C. (2005). Theta burst stimulation of the human motor cortex. Neuron, 45(2), 201–206. 10.1016/j.neuron.2004.12.033, 15664172

[bib55] Humphries, C., Love, T., Swinney, D., & Hickok, G. (2005). Response of anterior temporal cortex to syntactic and prosodic manipulations during sentence processing. Human Brain Mapping, 26(2), 128–138. 10.1002/hbm.20148, 15895428 PMC6871757

[bib56] Indefrey, P., Hellwig, F., Herzog, H., Seitz, R. J., & Hagoort, P. (2004). Neural responses to the production and comprehension of syntax in identical utterances. Brain and Language, 89(2), 312–319. 10.1016/S0093-934X(03)00352-3, 15068913

[bib57] Ishkhanyan, B., Michel Lange, V., Boye, K., Mogensen, J., Karabanov, A., Hartwigsen, G., & Siebner, H. R. (2020). Anterior and posterior left inferior frontal gyrus contribute to the implementation of grammatical determiners during language production. Frontiers in Psychology, 11, Article 685. 10.3389/fpsyg.2020.00685, 32395113 PMC7197372

[bib458] JASP Team. (2023). JASP (Version 0.17.1.0) [Software].

[bib58] Jodzio, A., Piai, V., Verhagen, L., Cameron, I., & Indefrey, P. (2023). Validity of chronometric TMS for probing the time-course of word production: A modified replication. Cerebral Cortex, 33(12), 7816–7829. 10.1093/cercor/bhad081, 37143175 PMC10267630

[bib59] Jung, J., & Lambon Ralph, M. A. (2021). The immediate impact of transcranial magnetic stimulation on brain structure: Short-term neuroplasticity following one session of cTBS. NeuroImage, 240, Article 118375. 10.1016/j.neuroimage.2021.118375, 34245868 PMC8456691

[bib60] Just, M. A., Carpenter, P. A., Keller, T. A., Eddy, W. F., & Thulborn, K. R. (1996). Brain activation modulated by sentence comprehension. Science, 274(5284), 114–116. 10.1126/science.274.5284.114, 8810246

[bib61] Kaan, E., & Swaab, T. Y. (2002). The brain circuitry of syntactic comprehension. Trends in Cognitive Sciences, 6(8), 350–356. 10.1016/S1364-6613(02)01947-2, 12140086

[bib62] *Kroczek, L. O. H., Gunter, T. C., Rysop, A. U., Friederici, A. D., & Hartwigsen, G. (2019). Contributions of left frontal and temporal cortex to sentence comprehension: Evidence from simultaneous TMS-EEG. Cortex, 115, 86–98. 10.1016/j.cortex.2019.01.010, 30776735

[bib63] Kuhl, P. K., Conboy, B. T., Padden, D., Nelson, T., & Pruitt, J. (2005). Early speech perception and later language development: Implications for the “critical period.” Language Learning and Development, 1(3–4), 237–264. 10.1080/15475441.2005.9671948

[bib64] *Kuhnke, P., Meyer, L., Friederici, A. D., & Hartwigsen, G. (2017). Left posterior inferior frontal gyrus is causally involved in reordering during sentence processing. NeuroImage, 148, 254–263. 10.1016/j.neuroimage.2017.01.013, 28069544

[bib65] Lim, H., & Godfroid, A. (2015). Automatization in second language sentence processing: A partial, conceptual replication of Hulstijn, Van Gelderen, and Schoonen’s 2009 study. Applied Psycholinguistics, 36(5), 1247–1282. 10.1017/S0142716414000137

[bib66] Liu, T.-H., Lai, C.-H., & Chou, T.-L. (2023). The neurocognitive basis of Chinese idiomatic constructions and processing differences between native speakers and L2 learners of Mandarin. Frontiers in Psychology, 14, Article 1112611. 10.3389/fpsyg.2023.1112611, 36910827 PMC9996060

[bib67] Makuuchi, M., Bahlmann, J., Anwander, A., & Friederici, A. D. (2009). Segregating the core computational faculty of human language from working memory. Proceedings of the National Academy of Sciences of the United States of America, 106(20), 8362–8367. 10.1073/pnas.0810928106, 19416819 PMC2688876

[bib68] Makuuchi, M., Grodzinsky, Y., Amunts, K., Santi, A., & Friederici, A. D. (2013). Processing noncanonical sentences in Broca’s region: Reflections of movement distance and type. Cerebral Cortex, 23(3), 694–702. 10.1093/cercor/bhs058, 22437052 PMC3563336

[bib69] Maran, M., Friederici, A. D., & Zaccarella, E. (2022). Syntax through the looking glass: A review on two-word linguistic processing across behavioral, neuroimaging and neurostimulation studies. Neuroscience and Biobehavioral Reviews, 142, Article 104881. 10.1016/j.neubiorev.2022.104881, 36210580

[bib70] *Maran, M., Numssen, O., Hartwigsen, G., & Zaccarella, E. (2022). Online neurostimulation of Broca’s area does not interfere with syntactic predictions: A combined TMS-EEG approach to basic linguistic combination. Frontiers in Psychology, 13, Article 968836. 10.3389/fpsyg.2022.968836, 36619118 PMC9815778

[bib71] *Maria-Korina, S., Elizabeth, C., Anastasios, B., Elias D., K., Arhonto, T., & Adamantia, M. (2015). The role of Broca’s area in syntax: A TMS study on written Greek language. European Scientific Journal, 2, 36–43. https://eujournal.org/index.php/esj/article/view/6479

[bib72] Matchin, W., Hammerly, C., & Lau, E. (2017). The role of the IFG and pSTS in syntactic prediction: Evidence from a parametric study of hierarchical structure in fMRI. Cortex, 88, 106–123. 10.1016/j.cortex.2016.12.010, 28088041

[bib73] *Meyer, L., Elsner, A., Turker, S., Kuhnke, P., & Hartwigsen, G. (2018). Perturbation of left posterior prefrontal cortex modulates top-down processing in sentence comprehension. NeuroImage, 181, 598–604. 10.1016/j.neuroimage.2018.07.059, 30055371

[bib74] Meyer, L., Obleser, J., Anwander, A., & Friederici, A. D. (2012). Linking ordering in Broca’s area to storage in left temporo-parietal regions: The case of sentence processing. NeuroImage, 62(3), 1987–1998. 10.1016/j.neuroimage.2012.05.052, 22634860

[bib75] Miyagawa, S., Berwick, R. C., & Okanoya, K. (2013). The emergence of hierarchical structure in human language. Frontiers in Psychology, 4, Article 71. 10.3389/fpsyg.2013.00071, 23431042 PMC3577014

[bib76] Moro, A. (2014). On the similarity between syntax and actions. Trends in Cognitive Sciences, 18(3), 109–110. 10.1016/j.tics.2013.11.006, 24370077

[bib77] Musso, M., Moro, A., Glauche, V., Rijntjes, M., Reichenbach, J., Büchel, C., & Weiller, C. (2003). Broca’s area and the language instinct. Nature Neuroscience, 6(7), 774–781. 10.1038/nn1077, 12819784

[bib78] O’Grady, W. (1997). Syntactic development. University of Chicago Press. 10.7208/chicago/9780226620787.001.0001

[bib79] Ohta, S., Fukui, N., & Sakai, K. L. (2013). Syntactic computation in the human brain: The degree of merger as a key factor. PLOS ONE, 8(2), Article e56230. 10.1371/journal.pone.0056230, 23437097 PMC3577822

[bib80] Pallier, C., Devauchelle, A.-D., & Dehaene, S. (2011). Cortical representation of the constituent structure of sentences. Proceedings of the National Academy of Sciences of the United States of America, 108(6), 2522–2527. 10.1073/pnas.1018711108, 21224415 PMC3038732

[bib81] Pestalozzi, M. I., Di Pietro, M., Martins Gaytanidis, C., Spierer, L., Schnider, A., Chouiter, L., Colombo, F., Annoni, J.-M., & Jost, L. B. (2018). Effects of prefrontal transcranial direct current stimulation on lexical access in chronic poststroke aphasia. Neurorehabilitation and Neural Repair, 32(10), 913–923. 10.1177/1545968318801551, 30269644

[bib82] Petersson, K.-M., Folia, V., & Hagoort, P. (2012). What artificial grammar learning reveals about the neurobiology of syntax. Brain and Language, 120(2), 83–95. 10.1016/j.bandl.2010.08.003, 20943261

[bib83] Pinet, S., & Nozari, N. (2021). The role of visual feedback in detecting and correcting typing errors: A signal detection approach. Journal of Memory and Language, 117, Article 104193. 10.1016/j.jml.2020.104193

[bib84] Pulvermüller, F., & Fadiga, L. (2010). Active perception: Sensorimotor circuits as a cortical basis for language. Nature Reviews Neuroscience, 11(5), 351–360. 10.1038/nrn2811, 20383203

[bib85] Qu, X., Wang, Z., Cheng, Y., Xue, Q., Li, Z., Li, L., Feng, L., Hartwigsen, G., & Chen, L. (2022). Neuromodulatory effects of transcranial magnetic stimulation on language performance in healthy participants: Systematic review and meta-analysis. Frontiers in Human Neuroscience, 16, Article 1027446. 10.3389/fnhum.2022.1027446, 36545349 PMC9760723

[bib86] Rogalsky, C., & Hickok, G. (2009). Selective attention to semantic and syntactic features modulates sentence processing networks in anterior temporal cortex. Cerebral Cortex, 19(4), 786–796. 10.1093/cercor/bhn126, 18669589 PMC2651476

[bib87] Rogalsky, C., & Hickok, G. (2011). The role of Broca’s area in sentence comprehension. Journal of Cognitive Neuroscience, 23(7), 1664–1680. 10.1162/jocn.2010.21530, 20617890

[bib88] Rogalsky, C., Matchin, W., & Hickok, G. (2008). Broca’s area, sentence comprehension, and working memory: An fMRI Study. Frontiers in Human Neuroscience, 2, Article 14. 10.3389/neuro.09.014.2008, 18958214 PMC2572210

[bib89] Roy, A. C., Curie, A., Nazir, T., Paulignan, Y., des Portes, V., Fourneret, P., & Deprez, V. (2013). Syntax at hand: Common syntactic structures for actions and language. PLOS ONE, 8(8), Article e72677. 10.1371/journal.pone.0072677, 23991140 PMC3749983

[bib90] *Sakai, K. L., Noguchi, Y., Takeuchi, T., & Watanabe, E. (2002). Selective priming of syntactic processing by event-related transcranial magnetic stimulation of Broca’s area. Neuron, 35(6), 1177–1182. 10.1016/S0896-6273(02)00873-5, 12354406

[bib91] Santi, A., & Grodzinsky, Y. (2007a). Taxing working memory with syntax: Bihemispheric modulations. Human Brain Mapping, 28(11), 1089–1097. 10.1002/hbm.20329, 17133392 PMC6871416

[bib92] Santi, A., & Grodzinsky, Y. (2007b). Working memory and syntax interact in Broca’s area. NeuroImage, 37(1), 8–17. 10.1016/j.neuroimage.2007.04.047, 17560794

[bib93] Santi, A., & Grodzinsky, Y. (2010). fMRI adaptation dissociates syntactic complexity dimensions. NeuroImage, 51(4), 1285–1293. 10.1016/j.neuroimage.2010.03.034, 20338244 PMC2909752

[bib94] Schell, M., Zaccarella, E., & Friederici, A. D. (2017). Differential cortical contribution of syntax and semantics: An fMRI study on two-word phrasal processing. Cortex, 96, 105–120. 10.1016/j.cortex.2017.09.002, 29024818

[bib95] Schuhmann, T., Schiller, N. O., Goebel, R., & Sack, A. T. (2009). The temporal characteristics of functional activation in Broca’s area during overt picture naming. Cortex, 45(9), 1111–1116. 10.1016/j.cortex.2008.10.013, 19111289

[bib96] Segalowitz, N. [S.], & Hulstijn, J. (2005). Automaticity in bilingualism and second language learning. In J. F. Kroll & A. M. B. De Groot (Eds.), Handbook of bilingualism: Psycholinguistic approaches (pp. 371–388). Oxford University Press. 10.1093/oso/9780195151770.003.0021

[bib97] Segalowitz, N. S., & Segalowitz, S. J. (1993). Skilled performance, practice, and the differentiation of speed-up from automatization effects: Evidence from second language word recognition. Applied Psycholinguistics, 14(3), 369–385. 10.1017/S0142716400010845

[bib98] Sliwinska, M. W., Elson, R., & Pitcher, D. (2021). Stimulating parietal regions of the multiple-demand cortex impairs novel vocabulary learning. Neuropsychologia, 162, Article 108047. 10.1016/j.neuropsychologia.2021.108047, 34610342

[bib99] Stanislaw, H., & Todorov, N. (1999). Calculation of signal detection theory measures. Behavior Research Methods, Instruments, & Computers, 31(1), 137–149. 10.3758/BF03207704, 10495845

[bib100] Steel, A., Song, S., Bageac, D., Knutson, K. M., Keisler, A., Saad, Z. S., Wasserman, E. M., & Wilkinson, L. (2016). Shifts in connectivity during procedural learning after motor cortex stimulation: A combined transcranial magnetic stimulation/functional magnetic resonance imaging study. Cortex, 74, 134–148. 10.1016/j.cortex.2015.10.004, 26673946 PMC4724496

[bib101] Stout, D., & Chaminade, T. (2009). Making tools and making sense: Complex, intentional behaviour in human evolution. Cambridge Archaeological Journal, 19(1), 85–96. 10.1017/S0959774309000055

[bib102] Sun, X., Hancock, R., Bever, T. G., Cheng, X., Schmidt, L., & Seifert, U. (2016). Processing relative clause in Chinese: Evidence from event-related potentials. Chinese Journal of Applied Linguistics, 39(1), 92–114. 10.1515/cjal-2016-0006

[bib103] Sun, Z., Shi, Y., Guo, P., Yang, Y., & Zhu, Z. (2021). Independent syntactic representation identified in left front-temporal cortex during Chinese sentence comprehension. Brain and Language, 214, Article 104907. 10.1016/j.bandl.2021.104907, 33503520

[bib104] Thibault, S., Py, R., Gervasi, A. M., Salemme, R., Koun, E., Lövden, M., Boulenger, V., Roy, A. C., & Brozzoli, C. (2021). Tool use and language share syntactic processes and neural patterns in the basal ganglia. Science, 374(6569), Article eabe0874. 10.1126/science.abe0874, 34762470

[bib105] Tolentino, L. C., & Tokowicz, N. (2014). Cross-language similarity modulates effectiveness of second language grammar instruction. Language Learning, 64(2), 279–309. 10.1111/lang.12048

[bib106] Tyler, L. K., Shafto, M. A., Randall, B., Wright, P., Marslen-Wilson, W. D., & Stamatakis, E. A. (2010). Preserving syntactic processing across the adult life span: The modulation of the frontotemporal language system in the context of age-related atrophy. Cerebral Cortex, 20(2), 352–364. 10.1093/cercor/bhp105, 19505991 PMC2803734

[bib107] *Uddén, J., Folia, V., Forkstam, C., Ingvar, M., Fernandez, G., Overeem, S., van Elswijk, G., Hagoort, P., & Petersson, K. M. (2008). The inferior frontal cortex in artificial syntax processing: An rTMS study. Brain Research, 1224, 69–78. 10.1016/j.brainres.2008.05.070, 18617159

[bib108] *Uddén, J., Ingvar, M., Hagoort, P., & Petersson, K. M. (2017). Broca’s region: A causal role in implicit processing of grammars with crossed non-adjacent dependencies. Cognition, 164, 188–198. 10.1016/j.cognition.2017.03.010, 28453996

[bib109] *van der Burght, C. L., Numssen, O., Schlaak, B., Goucha, T., & Hartwigsen, G. (2023). Differential contributions of inferior frontal gyrus subregions to sentence processing guided by intonation. Human Brain Mapping, 44(2), 585–598. 10.1002/hbm.26086, 36189774 PMC9842926

[bib110] Wang, P., Knösche, T. R., Chen, L., Brauer, J., Friederici, A. D., & Maess, B. (2021). Functional brain plasticity during L1 training on complex sentences: Changes in gamma-band oscillatory activity. Human Brain Mapping, 42(12), 3858–3870. 10.1002/hbm.25470, 33942956 PMC8288093

[bib111] Wang, S., Zhu, Z., Zhang, J. X., Wang, Z., Xiao, Z., Xiang, H., & Chen, H.-C. (2008). Broca’s area plays a role in syntactic processing during Chinese reading comprehension. Neuropsychologia, 46(5), 1371–1378. 10.1016/j.neuropsychologia.2007.12.020, 18255103

[bib112] Ward, E., Brownsett, S. L. E., McMahon, K. L., Hartwigsen, G., Mascelloni, M., & de Zubicaray, G. I. (2022). Online transcranial magnetic stimulation reveals differential effects of transitivity in left inferior parietal cortex but not premotor cortex during action naming. Neuropsychologia, 174, Article 108339. 10.1016/j.neuropsychologia.2022.108339, 35921869

[bib113] Wischnewski, M., & Schutter, D. J. (2015). Efficacy and time course of theta burst stimulation in healthy humans. Brain Stimulation, 8(4), 685–692. 10.1016/j.brs.2015.03.004, 26014214

[bib114] Wu, C.-Y., Zaccarella, E., & Friederici, A. D. (2019). Universal neural basis of structure building evidenced by network modulations emerging from Broca’s area: The case of Chinese. Human Brain Mapping, 40(6), 1705–1717. 10.1002/hbm.24482, 30468022 PMC6865561

[bib115] Xu, K., Wu, D. H., & Duann, J.-R. (2020a). Dynamic brain connectivity attuned to the complexity of relative clause sentences revealed by a single-trial analysis. NeuroImage, 217, Article 116920. 10.1016/j.neuroimage.2020.116920, 32422404

[bib116] Xu, K., Wu, D. H., & Duann, J.-R. (2020b). Enhanced left inferior frontal to left superior temporal effective connectivity for complex sentence comprehension: fMRI evidence from Chinese relative clause processing. Brain and Language, 200, Article 104712. 10.1016/j.bandl.2019.104712, 31704517

[bib117] Yang, C. L., Perfetti, C. A., & Liu, Y. (2010). Sentence integration processes: An ERP study of Chinese sentence comprehension with relative clauses. Brain and Language, 112(2), 85–100. 10.1016/j.bandl.2009.10.005, 20006378

[bib118] Zaccarella, E., & Friederici, A. D. (2015). Merge in the human brain: A sub-region based functional investigation in the left pars opercularis. Frontiers in Psychology, 6, Article 1818. 10.3389/fpsyg.2015.01818, 26640453 PMC4661288

[bib119] Zaccarella, E., Papitto, G., & Friederici, A. D. (2021). Language and action in Broca’s area: Computational differentiation and cortical segregation. Brain and Cognition, 147, Article 105651. 10.1016/j.bandc.2020.105651, 33254030

[bib120] Zaccarella, E., Schell, M., & Friederici, A. D. (2017). Reviewing the functional basis of the syntactic merge mechanism for language: A coordinate-based activation likelihood estimation meta-analysis. Neuroscience and Biobehavioral Reviews, 80, 646–656. 10.1016/j.neubiorev.2017.06.011, 28743620

[bib121] Zhu, D. X. (1985). The questions and answers on grammar. Commercial Press.

[bib122] Zhu, Y., Xu, M., Lu, J., Hu, J., Kwok, V. P. Y., Zhou, Y., Yuan, D., Wu, B., Zhang, J., Wu, J., & Tan, L. H. (2022). Distinct spatiotemporal patterns of syntactic and semantic processing in human inferior frontal gyrus. Nature Human Behavior, 6(8), 1104–1111. 10.1038/s41562-022-01334-6, 35618778

