# Supplementary material for: Continuous Theta-Burst Stimulation on the Left Posterior Inferior Frontal Gyrus Perturbs Complex Syntactic Processing Stability in Mandarin Chinese
Source: Neurobiol Lang (Camb). 2024 Jun 24;5(2):608–27. doi: 10.1162/nol_a_00140 (PMC11210936; doi:10.1162/nol_a_00140)
Supplement: Supplementary file 1 [file nol-5-2-608-s001.pdf]

Supporting Information

Continuous Theta-Burst Stimulation on the Left Posterior Inferior Frontal Gyrus Perturbs Complex Syntactic Processing Stability in Mandarin Chinese

Accuracy Analysis Results

We adopted accuracy as a valuable behavioral index to assess whether certain sequence types/tasks showed ceiling effects leading to that variability in performance may be greater as a function of task difficulty independent of nature of the task. Descriptive statistics were shown in Table S1, and plotted in Figure S1.

Since the accuracy of the easiest task, that is, the simple sentence processing, did not reach 90%, we assumed that none of the tasks showed a ceiling effect. Moreover, two-way repeated measures ANOVA was performed for comparing either the 3 (Complex, Simple, Word List) or the 4 sequence types (SR, OR, Simple, Word List). Results showed that only the main effect of sequence types was significant [for 3 sequence types: $F(2, 62) = 52.472$, $p < .001$, $\eta_p^2 = .629$; for 4 sequence types: $F(3, 93) = 34.502$, $p < .001$, $\eta_p^2 = .527$]. *Post hoc* paired comparisons showed that the accuracy of simple sentence was significantly higher than those of both complex (SR and OR) sentence and word list processing conditions [for 3 sequence types: ts(31) ≥ 8.670, p_{bonf}s < .001, *Cohen's d*s ≥ 1.275; for 4 sequence types: ts(31) ≥ 6.172, p_{bonf}s < .001, *Cohen's d*s ≥ .828]. Nevertheless, no statistical accuracy differences could be found between the complex (SR and OR) sentence and word list processing conditions [Complex vs. Word List: $t(31) = .068$, $p_{bonf} = 1.000$, *Cohen's d* = .009; SR vs.

Word List: $t(31) = 1.860$, $p_{bonf} = .435$, *Cohen's d* = .254; OR vs. Word List: $t(31) = -2.148$, $p_{bonf} = .238$, *Cohen's d* = .329]. The accuracy difference pattern was also consistent with the results reported in a previous fMRI study (Liu et al., 2023: *Supporting Information 1.2*), in which the difficulty of the word list processing (i.e., the working memory) task was well matched with that of the complex sentence (either SR or OR) processing task. Therefore, the cTBS effect difference between complex sentence and word list processing conditions was more likely to be attributed to the nature of the task *per se* rather than the difficulty difference between these two tasks.

Table S1

Sequence type accuracy under cTBS and sham conditions

Sequence types	Stimulation conditions	Mean	SD
Complex (C)	cTBS	0.744	0.123
	sham	0.753	0.114
OR	cTBS	0.702	0.136
	sham	0.714	0.146
SR	cTBS	0.786	0.135
	sham	0.771	0.158
Simple (S)	cTBS	0.881	0.068
	sham	0.877	0.078
Word List (W)	cTBS	0.740	0.115
	sham	0.755	0.105

Figure S1

Raincloud plots for accuracy of the 3 and 4 sequence types under both cTBS and sham conditions

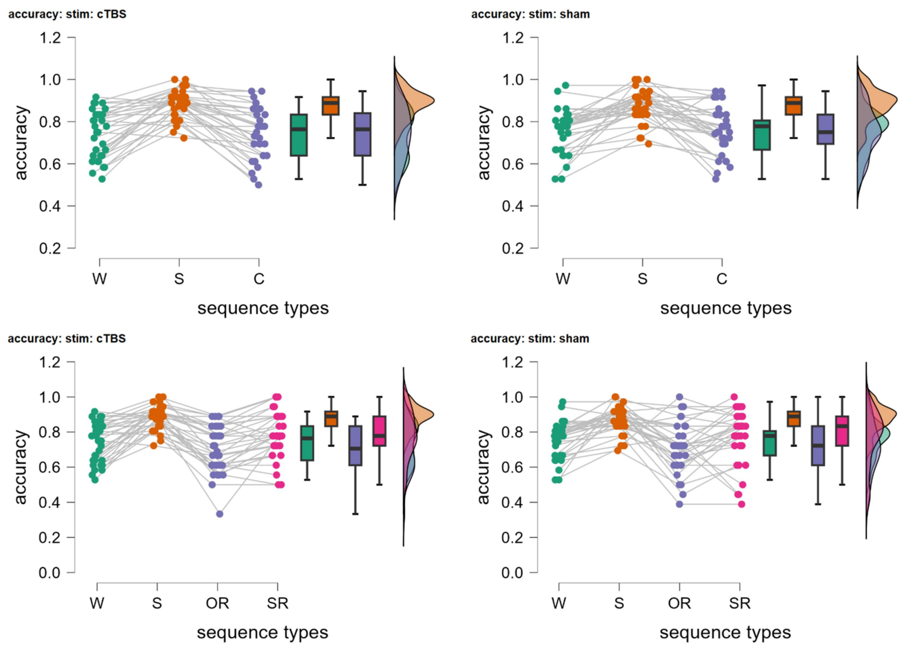

Note. W: word list processing (i.e., working memory task), colored in green; S: simple sentence processing, colored in orange; C: complex sentence processing, colored in purple. SR: complex sentence with subject relative clause embedded processing, colored in pink; OR: complex sentence with object relative clause embedded processing, colored in purple.

Further Validation of the cTBS Effect Robustness

Behavioral Data Analysis Blind to the Conditions

Given that we intended to compare the differences ("Δ": cTBS - sham) between the conditions (either 3 or 4 sequence types), experimenters were not bind to the sessions (cTBS or sham) during the data analyses as described in the main text. A concern was whether the blindness to the data/conditions would have biased the results. Therefore, as an *ad-hoc* test,

we invited another experimenter who was totally blind to the conditions to re-analyze the *CV*(coefficient of variation) data which showed significance between the three/four conditions. In particular, the cTBS condition was masked as the "*a*" condition, and the sham condition was labeled as "*α*", and the experimenter was asked to subtract the *CV* data of one condition from the other according to his own will. The experimenter obtained the *ΔCV* from the subtraction of "*a* - *α*", and the repeated measures ANOVA showed exactly the same results to those reported in the main text, thus indicating that in the present study, the blindness to the conditions had little bias to the current analyses as well as the related results.

Behavioral Data Analyses of the Whole Set of Participants

We originally recruited 33 participants in total. However, there was one more participant who underwent the sham session firstly, resulting in the unbalanced numbers of the two stimulation types for each session: "17 sham + 16 cTBS" for the first session, and "16 sham + 17 cTBS" for the second session. Therefore, before the actual data analyses, data of one of the participants who attended the sham session as the first session was randomly discarded. Now we added this participant's data for the *ΔCV* analysis of the whole set of participants, and found the similar result in the comparison among the 4 sequence types [$F(3, 96) = 3.510$, $p=.018$, $\eta_p^2 = .099$] as reported in the main text, further indicating that the cTBS effect (reflected by the differences of *ΔCV* between the sequence types) should be robust.

Analysis of the Session Order Effect

To further assess the potential influence of session order effects, we divided the participants into two groups: Group 1 (subjects who received real cTBS first, then followed by "sham") and Group 2 (subjects who received "sham" first, followed by real cTBS). Then

we performed 3 (sequence types: Complex, Simple, and Word List) × 2 (group: Group 1 and Group 2) and 4 (sequence types: SR, OR, Simple, and Word List) × 2 (group: Group 1 and Group 2) ANOVAs separately on the indices *Δd'*, *ΔRT*, and *ΔCV*. In these ANOVAs, the sequence types were used as a with-subject variable while the group served as a between-subject variable. Descriptive statistics were generated for all indices and summarized in Table S2 to Table S4.

The results for *Δd'* showed that the two-way interactions were significant for 3 sequence types [$F(2, 60) = 6.783$, $p = .002$, $\eta_p^2 = .184$] and 4 sequence types [$F(2.259, 67.759) = 3.888$, $p = .021$, $\eta_p^2 = .115$]. These results indicated that the effect of sequence type on *Δd'* is different in Group 1 and Group 2. Nevertheless, there were no group main effects observed for both 3 sequence types [$F(1, 30) = .061$, $p = .806$, $\eta_p^2 = .002$] and 4 sequence types [$F(1, 30) = .753$, $p = .392$, $\eta_p^2 = 0.024$]. Similarly, there were no sequence type main effects observed for both 3 sequence types [$F(2, 60) = .490$, $p = .615$, $\eta_p^2 = .016$] and 4 sequence types [$F(2.614, 67.759) = 1.303$, $p = .280$, $\eta_p^2 = .042$]. Therefore, when combining the two groups' data together, the session order effect on *Δd'* would be "neutralized".

The results for *ΔRT* showed no significant group differences in both the 3 sequence types [$F(1, 30) = 1.154$, $p = .291$, $\eta_p^2 = .037$] and the 4 sequence types [$F(1, 30) = 1.754$, $p = .195$, $\eta_p^2 = .055$]. Additionally, there were no significant two-way interactions observed for both the 3 sequence types [$F(2, 60) = 1.439$, $p = .245$, $\eta_p^2 = .046$] and the 4 sequence types [$F(3, 90) = 1.025$, $p = .385$, $\eta_p^2 = .033$]. Moreover, there were no sequence type main effects observed for both the 3 sequence types [$F(2, 60) = 1.041$, $p = .359$, $\eta_p^2 = .034$] and the 4 sequence types [$F(3, 90) = 0.932$, $p = .429$, $\eta_p^2 = .030$].

The results for *ΔCV* showed no significant group differences in the 3 sequence types [$F(1, 30) = 4.790 \times 10^{-4}$, $p = .983$, $\eta_p^2 = 1.597 \times 10^{-5}$] or the 4 sequence types [$F(1, 30) = .068$, $p = .796$, $\eta_p^2 = .002$]. Additionally, there were no significant two-way interactions observed in the 3 sequence types [$F(2, 60) = .620$, $p = .542$, $\eta_p^2 = .020$] or the 4 sequence types [$F(3, 90) = .547$, $p = .651$, $\eta_p^2 =. 018$]. Only the sequence types showed significant main effects in both the 3 sequence types [$F(2, 60) = 3{,}419$, $p = .039$, $\eta_p^2 = .012$] and the 4 sequence types [$F(3, 90) = 3.975$, $p = .010$, $\eta_p^2 = .117$].

Therefore, the session order was unlikely to affect the stimulation effect differences among the sequence types.

Table S2

Sequence type Δd' under Group 1 and Group 2

**Sequence types**	**Stimulation conditions**	**Mean**	**SD**
Complex (C)	Group1	-0.222	0.707
	Group2	0.077	0.614
OR	Group1	-0.392	1.129
	Group2	-0.093	0.947
SR	Group1	-0.053	0.533
	Group2	0.247	0.731
Simple (S)	Group1	-0.155	0.423
	Group2	0.367	0.680
**Sequence types**	**Stimulation**	**Mean**	**SD**

	conditions		
Word List (W)	Group1	0.422	0.758
	Group2	-0.326	0.736

Note. Group 1 contained subjects who received the real cTBS condition followed by the “sham” condition and Group 2 contained subjects who received the “sham” condition followed by the real cTBS condition.

Table S3

Sequence type ΔRT under Group 1 and Group 2

**Sequence types**	**Stimulation conditions**	**Mean**	**SD**
Complex (C)	Group1	11.546	124.843
	Group2	-43.101	93.126
OR	Group1	-2.663	113.639
	Group2	-44.807	118.228
SR	Group1	25.755	162.358
	Group2	-43.368	140.120
Simple (S)	Group1	-8.663	117.969
	Group2	18.161	127.761
Word List (W)	Group1	48.368	96.601
	Group2	-1.975	105.566

Note. Group 1 contained subjects who received the real cTBS condition followed by the "sham" condition and Group 2 contained subjects who received the "sham" condition followed by the real cTBS condition.

Table S4

Sequence type ΔCV under Group 1 and Group 2

**Sequence types**	**Stimulation conditions**	**Mean**	**SD**
Complex (C)	Group1	0.019	0.045
	Group2	0.030	0.039
OR	Group1	0.010	0.070
	Group2	0.012	0.054
SR	Group1	0.027	0.054
	Group2	0.047	0.046
Simple (S)	Group1	-0.003	0.081
	Group2	-0.023	0.071
Word List (W)	Group1	-0.012	0.066
	Group2	-0.004	0.047

Note. Group 1 contained subjects who received the real cTBS condition followed by the "sham" condition and Group 2 contained subjects who received the "sham" condition followed by the real cTBS condition.

SI References

Liu, Y., Gao, C., Wang, P., Friederici, A. D., Zaccarella, E., & Chen, L. (2023). Exploring the neurobiology of Merge at a basic level: insights from a novel artificial grammar paradigm. *Frontiers in Psychology*, 14, 1151518. https://doi.org/10.3389/fpsyg.2023.1151518
